# Revisiting the Body-Schema Concept in the Context of Whole-Body Postural-Focal Dynamics

**DOI:** 10.3389/fnhum.2015.00083

**Published:** 2015-02-17

**Authors:** Pietro Morasso, Maura Casadio, Vishwanathan Mohan, Francesco Rea, Jacopo Zenzeri

**Affiliations:** ^1^Robotics, Brain and Cognitive Sciences Department, Istituto Italiano di Tecnologia, Genoa, Italy; ^2^Dipartimento di Informatica, Bioingegneria, Robotica e Ingegneria dei Sistemi (DIBRIS), University of Genoa, Genoa, Italy

**Keywords:** body schema, whole-body movements, passive motion paradigm, internal models, embodied cognition, synergy formation, equilibrium-point hypothesis

## Abstract

The body-schema concept is revisited in the context of embodied cognition, further developing the theory formulated by Marc Jeannerod that the motor system is part of a simulation network related to action, whose function is not only to shape the motor system for preparing an action (either overt or covert) but also to provide the self with information on the feasibility and the meaning of potential actions. The proposed computational formulation is based on a dynamical system approach, which is linked to an extension of the equilibrium-point hypothesis, called Passive Motor Paradigm: this dynamical system generates goal-oriented, spatio-temporal, sensorimotor patterns, integrating a direct and inverse internal model in a multi-referential framework. The purpose of such computational model is to operate at the same time as a general synergy formation machinery for planning whole-body actions in humanoid robots and/or for predicting coordinated sensory–motor patterns in human movements. In order to illustrate the computational approach, the integration of simultaneous, even partially conflicting tasks will be analyzed in some detail with regard to postural-focal dynamics, which can be defined as the fusion of a focal task, namely reaching a target with the whole-body, and a postural task, namely maintaining overall stability.

## The Origin of the Body-Schema Concept

The English neurologist Henry Head is probably the first who defined and used the term “body schema” (Head, 1920), further elaborating previous studies (Head and Holmes, 1911) on sensory disturbances from cerebral lesions. In this view of classical neurology, the brain holds a constantly updated status of the body shape and posture as an ongoing, mainly unconscious, integration of successive proprioceptive signals, somehow distinct from a more conscious representation of the body, or body image (Gallagher, 1998).

Modern neuroscience as well as cybernetics has greatly enriched and expanded the concept. In particular, it has become clear that different sensory modalities, in addition to proprioception, are integrated in such process (Graziano and Botvinick, 1999; Armel and Ramachandran, 2003; Maravita and Iriki, 2004). Neurophysiological recordings demonstrated that such internal body representation is also involved in action (Jeannerod et al., 1995; Berlucchi and Aglioti, 1997; Rizzolatti et al., 1997; Colby, 1998; Graziano and Gross, 1998). Recent studies are also beginning to highlight, on one hand, the coupling between motor intentions and the internal representation of the body, and, on the other, the link between overt and covert actions. Consider, for example, that in patients undergoing awake brain surgery (Desmurget et al., 2009), electrical stimuli applied to inferior parietal regions evoke a strong intention to move the contralateral limb and the false belief that the movement was actually performed, whereas it was not; moreover, stimulation of premotor areas triggers indeed overt limb movements but the patients are unaware of it. From such experimental evidence, Desmurget and Sirigu (2009) proposed a parietal–premotor network model for movement intention and motor awareness that, in our view, incorporates part of the body schema.

In summary, we view the body schema as a set of fronto-parietal networks that integrate information originating from regions of the body and external space in a way, which is functionally relevant to specific actions performed by different body parts. As such, the body schema is a representation of the body’s spatial properties, including the length of limbs and limb segments, their arrangement, the configuration of the segments in space, and the shape of the body surface; it can also incorporate, after suitable training, the spatial/dynamic properties of tools employed by skilled users (Maravita and Iriki, 2004). As a constantly updated postural model that keeps track of limb position, it plays an important role in the control of action and it involves both central and peripheral neural systems. In this framework, the contribution of cybernetics (in its different aspects, as dynamic systems theory, neural network theory, control theory, etc.) is basically to provide a (mathematical) language for representing and understanding the complexity of the dynamic interactions of neuromuscular/neurocognitive processes and to make predictions based on the simulation of the models. Thus, the body schema can be characterized by the following computational features:

- It is *spatially encoded*, in the sense that it represents both position and configuration of the body as a three-dimensional object in space (Macaluso and Maravita, 2010);
- It is *distributed and modular* (Haggard and Wolpert, 2005), because it is not represented in a single region of the brain and it involves a number of interacting modules, as fronto-parietal loops;
- It is *intermodal/supramodal*, in the sense that it integrates proprioceptive and tactile information for maintaining a 3D body representation, variable in time, while preserving its functional identity; moreover, this representation is also modulated by other sensory channels, e.g., vision, as a function of specific task constraints and environmental conditions. This suggests that the body schema is somehow responsible of integrating the different sensory modalities in such a way to achieve an abstract or *amodal* representation (Haggard and Wolpert, 2005), while preserving an action-oriented overall coherence^1^;
- It is characterized by a *short-term plasticity* and reorganization on the timescale of seconds, as shown by the quick integration of tools into the body schema (Maravita and Iriki, 2004).

## Coding and Frames of Reference in Sensorimotor Cortical Maps: The Body Schema is Dynamic and Multi-Referential

The response properties of neurons in motor/premotor cortical areas have been correlated with a wide variety of behavioral and cognitive variables, starting with early work of Evarts (1968), looking both at geometric/kinematic variables in a Cartesian frame of reference (e.g., position, speed and acceleration of the end-effector) and variables in intrinsic, joint, or muscle oriented reference frames (e.g., joint angular positions or velocities, end-point force, and muscle tensions). In particular, Georgopoulos et al. (1986) coined the term of Population Coding for expressing the rather large spread of the receptive fields of the investigated neurons and the distributed nature of such coding. The problem is that such correlational studies can only provide very weak evidence about the specific code and frame of reference embodied in sensorimotor cortical maps. Indeed, in a recent study that systematically investigated a large workspace for reaching movements, solid evidence was found against a single coordinate system representation in the motor cortex, thus suggesting the possible coexistence of multiple reference frames (Wu and Hatsopoulos, 2006). Moreover, given the observed heterogeneity and ambiguity of neuronal responses (Churchland and Shenoy, 2007) suggested that it may be necessary to discard the notion that the key to understand the organization of sensorimotor cortical areas is to pursue the “holy grail” of movement parameters representation. In any case, there is a general agreement that the problem of maintaining the consistency among spatial information in cortical maps and fronto-parietal circuits for controlling body movements is closely related to the concept of body schema (Haggard and Wolpert, 2005).

An alternative to the theory of Population Coding of motor control is the equilibrium-point hypothesis (EPH), which is a dynamics-based approach where movements are not “coded” but are the consequence of the intrinsic dynamics of the body and neuromuscular system. This approach exploits the viscoelastic properties of the musculoskeletal system, i.e., the intrinsic force fields associated with muscle tissues, and solves motor control problems without computing the joint angles and joint torques explicitly (Feldman, 1966; Feldman and Levin, 1995) but allowing the neuromuscular system to determine the shift from an equilibrium point to the next one. This point-based dynamic approach was then extended by positing the existence of a moving neuromuscular force field (or a virtual trajectory control), for guiding indirectly end-point movements (Bizzi et al., 1984). A further generalization of EPH, named passive motion paradigm (PMP: Mussa Ivaldi et al., 1988), will be further analyzed in the following with the main goal of narrowing the gap between motor cognition and motor control by developing a common dynamical model for real (overt) and imagined (covert) movements.

The alternative between the theory of Population Coding and the extended EPH may be considered an example of the memory-time tradeoff in algorithmic design, more specifically the tradeoff between look-up tables and recalculation. Population code is a kind of look-up table or motor memory and it can generate actions by a kind of read-out mechanism, whereas EPH is a parallel, dynamical system that generates actions by way of recalculation or simulation. The former approach is apparently more intuitive and intrinsically quicker but scales up badly with motor complexity, namely the number of degrees of freedom involved in an action; the latter approach is somehow slower but is more naturally suited for dealing with the high-dimensionality of whole-body movements because dynamical systems are parallel.

The issue of the preferred reference frame for explaining the patterns of activity of neurons in sensorimotor cortical maps occurs also in the analysis of the kinematic invariances in visually guided reaching movements, namely straight paths and bell-shaped velocity profiles (Morasso, 1981; Abend et al., 1982). Again, although such invariances are more easily expressed in spatial variables, this correlational evidence only makes it conceivable that the motor cortex can employ a Cartesian frame in the process of trajectory formation not that this is the unique representation channel. The rationale of involving many different frames of reference in the planning and control of reaching motions was formulated by Paillard (1991) and, in particular, a hybrid visuo-kinesthetic frame of reference was proposed by Carrozzo and Lacquaniti (1994). In conclusion, we can say that the body schema is likely to utilize multiple reference frames, not a single, preferred one: in other words, it is *multi-referential*. However, we also suggest that proprioception is the “glue” that keeps the coherence of the body schema during its incessant changing over time as a function of actor environment interactions.

The sense of the previous statement is related to the fact that the sensorimotor spaces associated to the different reference frames (joint space, task space, constraint space, tool space, etc.) have quite different dimensionality. The joint space is the one with the highest dimensionality and thus is the skeleton (or glue) on which everything else (goals, constraints, obstacle, etc.) can be built^2^. One may feel that a multi-referential framework is somehow under-specified, for example, in the sense that it is unclear to define what makes a cortical region a specific reference area. However, this kind of specific (and static) characterization of different reference areas is unnecessary in a dynamic, multi-referential framework. What matters is the overall dynamics of the set of interacting modules that can dynamically change the apparent “leading frame” of a given area and the corresponding “code” in different tasks and environmental conditions.

## The Body Schema is a Type of Integrated Internal Model

The notion of body schema originated from neurology, whereas the notion of internal model comes from robotics/cybernetics (Wolpert et al., 1995; Kawato, 1999; Tanaka and Sejnowski, 2013; Pickering and Clark, 2014) and has a computational flavor, as a network of systems that mimic the behavior of internal/external natural processes. Although usually described with different languages, in our opinion the two concepts have a lot in common. Human beings need internal models because they are not purely reactive agents. They use perception for driving purposive actions, but the linkage between perception and action is complex (because the human body is complex), is not unique (because the human body is redundant), and is not unidirectional, as in purely reactive systems, but is bidirectional because the flow of afferent signals (perception) determines the flow of efferent signals (action) and is reflected back as a reafferent flow that in turns modifies ongoing perception. In other words, humans need internal models for simulating the interaction of their own body with the environment or for anticipating/predicting the interaction among other external animate/inanimate entities.

Usually, two varieties of internal models are considered: (a) *forward models*, which simulate the causal flow in the dynamics of an action by predicting state modification (for example, position and velocity of the body parts) as a function of a given efferent flow of motor commands; (b) *inverse models*, which exploit a more or less precise knowledge of the system’s dynamics in order to anticipate the motor control patterns that are necessary for producing a desired action. Both types of models have a predictive nature: in the former case, they anticipate the sensory consequences of a given action whereas, in the latter, they predict the motor commands required by a desired action. However, it is somehow ironical that in many cases *inverse models* are used in the context of *feedforward control*, whereas *forward models* are used as modules in a *feedback control* loop.

Although with different names, the notion that internal models are needed by the brain in order to preserve a kind of *perceptual homeostasis* – namely a general sense of stability of the outside world in face of incessantly changing sensory patterns – was quite present to the minds of researchers of the nineteenth century (Purkinje, 1825; von Helmholtz, 1866, among others). The terms used were *efference copy, corollary discharge*, and *reafference*. The idea is that when a subject performs a voluntary, goal-oriented action a copy of the motor commands delivered to the final common pathway (*efference copy*) is also sent to an internal forward model that is supposed to predict the sensory consequences of the action (*corollary discharge*). Such corollary discharge is then compared with the real sensory patterns (*reafference*), i.e., the sensory consequences of the motor command, thus informing the central nervous system (CNS) about how well the expected action matched its actual counterpart. Sherrington (1900) nicknamed the “reference copy” as “sensation of innervation” and criticized it because, at that time, it remained “unproven.” This was enough to “ostracize” the corollary discharge hypothesis for over 50 years, until the seminal work of von Holst and Mittelstaedt (1950) on the optokinetic nystagmus of the fly, quickly followed by a number of studies that provided convincing evidence of corollary discharge – in the compensation of Coriolis effect, in gaze stability, in the prediction of grip forces, etc. In any case, central to the theory of corollary discharge is the posited internal model that transforms the efference copy in the corollary discharge itself.

A potential use of corollary discharge and the associated forward model is to compensate the large physiological delay of afference that deteriorates the stability and performance of feedback control mechanisms: with the use of a forward model for internal feedback, the outcome of an action can be estimated and used before sensory feedback is available. This is computationally quite interesting, but its relevance should not be overstretched, considering it as a sort of universal paradigm. There are indeed a number of potential limitations of corollary discharge if we try to include, in its domain of application, large-scale motor control, in addition to specific motor cognitive processes like planning and mental reasoning/training. For example, if we deal with unstable tasks, prediction of the sensory consequences of motor commands is quite unreliable. Moreover, if we consider whole-body movements, which may also involve unknown loads, a prediction model that can address dynamics of the whole body + load is virtually impossible, whereas the corresponding prediction of the kinematic patterns is doable, as we shall elucidate in the following. Even with this kind of limitations, the notion of internal forward model is of strong theoretical use, although its existence and implementation in the CNS are still a topic of debate.

The twin companion of the forward internal model concept is the inverse model, which is supposed to solve two different problems, although frequently confused when people mix motor control and motor cognitive aspects: (1) inverse dynamics and (2) inverse kinematics.

Inverse dynamics, in spite of its name, is not an inverse, ill-posed problem in the mathematical sense. It consists of predicting the torque control patterns required by desired kinematics and the solution of this problem is unique, although it is quite heavy from the computational point of view: it grows quickly with an increasing number of degrees of freedom (Khatib et al., 2004)^3^. Moreover, the anthropometric/robometric parameters that need to be specified, whether one follows the Newton–Euler or the Lagrange formalism, are difficult to evaluate and can change suddenly in the course of an action, for example, when manipulating objects or tools.

Differently from inverse dynamics, inverse kinematics is a typical example of ill-posed inverse mathematical problems that can be associated to motor redundancy, i.e., what is known as the “Degrees of Freedom Problem” (Bernstein, 1935). This is the computational process by which the brain coordinates the action of a high-dimensional set of motor variables for carrying out the tasks of everyday life, typically described and learnt in a task space of much lower dimensionality: such dimensionality imbalance is usually expressed by the term “motor redundancy,” meaning that the same movement goal can be achieved by an infinite number of combinations of the control variables, which are equivalent as far as the task is concerned. But in spite of so much freedom, experimental evidence suggests that the motor system consistently uses a narrow set of solutions. Consider, for example, the task of reaching a point B in space, starting from a point A, in a given time T. In principle, the task could be carried out in an infinite number of ways, with regards to spatial aspects (hand path), timing aspects (speed profile of the hand), and recruitment patterns of the available DoF’s (Degrees of Freedom). However, it is rather surprising that the spatio-temporal structure of this class of movements is strongly stereotypical, whatever their amplitude, direction, duration, and loading conditions: the path is nearly straight (in the extrinsic, Cartesian space, not the intrinsic, articulatory space) and the speed profile is nearly bell-shaped, with symmetric acceleration and deceleration phases (Morasso, 1981).

Where are such stereotypical patterns coming from and how is the corresponding inverse kinematic problem resolved? The typical robotic approach to inverse kinematics in redundant kinematic systems is to formulate it as an optimization problem. This requires defining a “cost function,” namely a mathematical combination of the control variables that is generally composed of two parts: a part that measures the “distance” of the current state from the goal state and a part (regularization term) that encodes the required “effort.” Starting with the early model of Flash and Hogan (1985), which used “integrated jerk” as the regularization term of the cost function, many other alternatives were proposed, such as “integrated torque change” (Uno et al., 1989), “end-point variance” (Harris and Wolpert, 1998), “object crackle” (Dingwell et al., 2004), or “acceleration criterion” (Ben-Itzhak and Karniel, 2008). The design is then reduced to the computation of the control variables that minimize the cost function, thus finding the best possible tradeoff between accuracy and effort. In all the cases, however, the spatio-temporal patterns generated by such complex optimization process are approximately the same and are compatible with the empirical patterns described above. This means that cost functions are very weak indicators of the stereotypical structure of goal-oriented movements. An alternative to the optimal control approach is a dynamics-based approach, named PMP (Mussa Ivaldi et al., 1988; Mohan and Morasso, 2011), which generalizes EPH, as is further explained in the following.

In general, forward and inverse internal models are usually considered separately. In contrast, we believe that they are two sides of the same predictive/control mechanism, namely the body schema, which plays the role of a computational middleware between motor cognition and motor control. While the latter is more strongly involved in inverse dynamics than inverse kinematics, the former is not necessarily involved in inverse dynamics, but quite strongly in inverse kinematics.

## The Body-Schema Concept and Embodied/Embedded Cognition

According to embodied cognition, our body, in all its aspects (sensory, motor, and body–environment interaction), shapes and organizes our mind, including high-level features (like memory, concepts, and categories) and abstract tasks (like reasoning and judgment). This unitary formulation of the body–mind system is clearly opposed to various forms of dualism, from the old-fashioned Cartesian dualism to more recent theories like cognitivism and conventional artificial intelligence (GOFAI: Good Old-Fashioned Artificial Intelligence). Some of the people who oppose GOFAI, like Brooks (1991), go the extreme of advocating a totally bottom-up architecture that is supposed to achieve “intelligence without representation”: this architecture is organized in layers, decomposing complicated intelligent behaviors into many “simple” behavioral modules, which in turn are organized into layers of simpler behaviors, down to reflex-like mechanisms. Ironically, such reductionist attitude of some modern roboticists is quite in tune with the general attitude of Charles Sherrington, who defended the theory that reflexes are the basic modules of the integrative action of the nervous system, thus enabling the entire body to function toward one definite goal at a time (Sherrington, 1906).

The main problem of layered bottom-up architectures is that they scale-up badly when one attempts to deal with complex bodies and complex behaviors in a complex environment. Thus, while maintaining an embodied cognitive attitude, we are not necessarily forced to the extreme of some form of reactive architecture but we can develop a cognitive neural architecture based on some kind of internal representation such as the body schema. As a matter of fact, in contrast with the Sherringtonian view, Liepmann (1905) was the first one to suggest that purposive actions are generated from within, requiring the existence of an internal state where they would be encoded, stored, and ultimately performed independently of the stimuli coming from the external environment. To account for the implementation of action plans, he proposed that the elementary chunks of action are assembled according to an internal representation: he called “movement formula” the result of this process, i.e., an anticipatory hierarchical structure where all the aspects of an action are represented, before it is enfolded in time. Liepmann’s legacy is still quite influential in motor neuroscience, although the term “movement formula” was later replaced by several others, like “engram,” “schema,” or “internal model,” and is clearly linked to the notion of embodied cognition.

According to Wilson (2002), embodied cognition is characterized by the following main features: (1) *cognition is situated*, in the sense that it is an online process, which takes place in the context of task-relevant sensorimotor information; (2) *cognition is time pressured*, i.e., it is constrained by the requirements of real-time interaction with the environment; (3) *the environment is part of the cognitive system*, including both the physical and social environment; (4) *cognition is intrinsically action oriented* and even “*off-line cognition*,” namely cognition without overt action, is bodily based in relation with a number of cognitive skills such as mental imagery, different forms of memory, reasoning, and problem-solving. Moreover, we can add that embodied cognition is spatially and topologically encoded.

For embodied cognition, the motor system may influence cognitive states and the latter may affect bodily actions: this is reflected in a large body of experimental evidence related to language acquisition, comprehension, and production (Glenberg and Gallese, 2012; Pickering and Garrod, 2013). Along the same line is the motor theory of speech perception (Liberman and Mattingly, 1985). The analysis of metaphors provides additional evidence in this direction, as shown by Lakoff and Johnson (1980): indeed, humans use metaphors ubiquitously and metaphors operate at a conceptual level, mapping one conceptual domain onto another. More generally, this expresses the strong feeling of the unitary nature and complementarity of “Perception and Action” that was proposed by Berthoz (2002), who focused on the crucial role of proprioception and kinesthesia in maintaining balance, coordinating actions, and navigating in a complex, structured world. The bidirectional relationship between the body and high-level features of the mind, such as self-consciousness and emotional states, has also been investigated by several people (Damasio, 1999; Gallagher, 2005, among the others).

In addition to the above-mentioned unitary nature and complementarity of “Perception and Action,” we should also consider the unitary nature and complementarity of “Action and Action-Observation” provided by the fronto-parietal mirror circuit in the cerebral cortex (Rizzolatti et al., 1997; Rizzolatti and Sinigaglia, 2010). A crucial development in this embodied cognitive framework was given, in our opinion, by Jeannerod (2001) who formulated the *Mental Simulation Theory*: it posits that cognitive motor processes such as motor imagery, movement observation, action planning, and verbalization share the same representations with motor execution and are implemented by running an internal model of the body schema. Jeannerod interpreted this brain activity as an internal simulation of a detailed representation of action and used the term S-state for describing the corresponding time-varying mental states. The crucial point is that since S-states occurring during covert actions are, to a great extent, quite similar to the states occurring during overt actions, then it is not unreasonable to posit that also real, overt actions are the results of the same internal simulation process, operating on an internal schematization of the body, i.e., a body schema. A further conclusion, suggested by such similarity, is that the posited internal simulation process is not directly involved with muscle contraction or biomechanics, shortly stated is “muscleless” and “massless.” Jeannerod stopped short of translating his theory into a computational model capable of running simulations at a high level of complexity. This is the subject of the next two sections of the paper.

In any case, from different directions, such as brain imaging studies, mirror neuron systems, and embodied cognition, there is mounting evidence that action generation, observation, imagination, and understanding share similar functional networks in the brain: distributed, multi-center neural activities occur not only during imagination of movement but also during observation and imitation of other’s actions and comprehension of language, namely action-related verbs and nouns. Such neural activation patterns include premotor and motor areas as well as areas of the cerebellum and the basal ganglia. During the observation of movements of others, an entire network of cortical areas, called “action-observation network,” is activated in a highly reproducible fashion. The central hypothesis that emerges out of these results is that motor imagery and motor cognition draw on a shared set of cortical and sub-cortical mechanisms underlying motor cognition.

Running internal simulations of overt/covert actions on an interconnected set of neuronal networks is, in our view, the main function of what is known as body schema. Therefore, the body schema must not be considered as a static structure, like the Penfield’s homunculus interpreted according to the common abused metaphor of the primary motor cortex as the keyboard of a piano, but as a non-linear dynamic system or pattern generator, like the Lorenz system or the Hodgkin–Huxley model, implemented in the neural circuitry in such a way to generate goal-oriented, spatio-temporal, sensorimotor patterns.

## A Formulation of the Body Schema Based on the PMP as a Synergy Formation Mechanism

**Figure 1:**
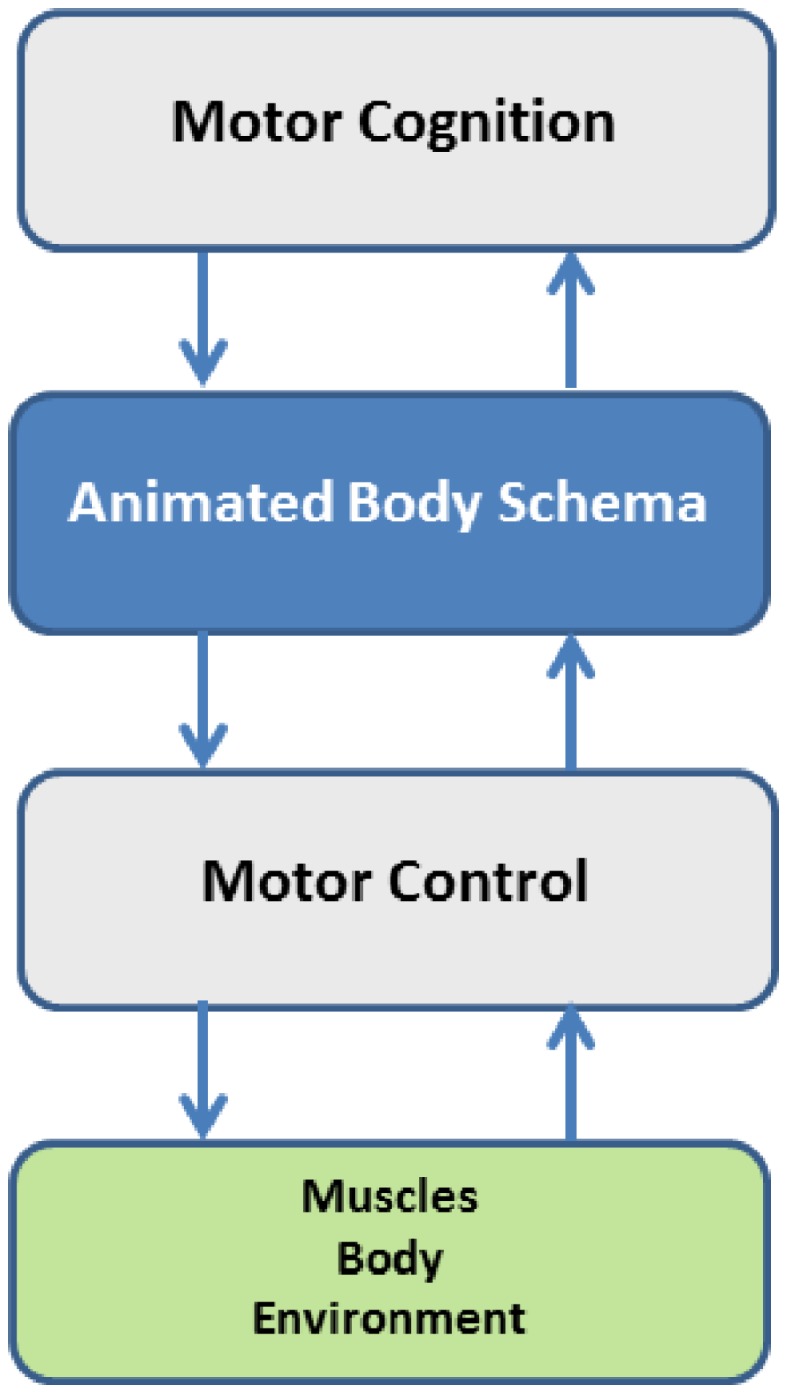
**The body schema as a computational middleware, separating motor cognition from motor control, but integrating them in the framework of embodiment**.

The proposal of the Body Schema as a computational model of the Mental Simulation Theory (Jeannerod, 2001) implies that it is a *middleware* between embodied motor cognition^4^ and motor control of the body (Figure 1). This means that the simulation or animation of the body schema is a task-oriented, multi-referential process that integrates multiple constraints in a parallel and distributed manner, thus introducing two important concepts in the analysis of the organization of action: one concept is the opportunity to separate motor cognition from motor control; the other concept is the identification of different time frames. The first concept is related to flexibility and the necessity of degrees of abstraction in the acquisition of skills. Mental reasoning and mental training can be powerful and effective only if it is possible to abstract from specific environmental conditions that can require different control strategies for the same task. The capability of abstraction is made possible by a body schema that allows formulating real and imagined actions in the same format. This logic separation of motor cognition and motor control implies the identification of three different time frames: (1) *learning time*, for acquiring an approximate representation of the model modules; (2) *preparation time*, for recruiting the necessary body parts, configuring the networks and setting up the specific task-dependent components; (3) *real-time*, for running the internal simulation of the body model and thus generating control patterns either for covert or overt actions.

The body schema, for its nature, incorporates the whole dimensionality of the body, but its task-oriented animation or synergy formation may involve a much smaller set of task-dependent variables. The concept of synergy, as a “dimensionality-reduction device,” was accompanied in early studies by the attempt to assign a regulatory role to the “spring-like” behavior of muscles (Bernstein, 1935) when such springiness was indeed evaluated by several experimental studies in the 60s and 70s (Asatryan and Feldman, 1965; Bizzi and Polit, 1978, among others). The central idea was that there is no chance in trying to explain biological movement in terms of engineering servomechanism theory, an approach supported for example by Marsden et al. (1972), first of all because muscles are not force/torque generators like electrical motors, but mainly because the propagation delays in the feedback loop are a severe, potential source of instability. In contrast, intrinsic muscle stiffness has two strong beneficial effects: (1) it provides, locally (i.e., in a muscle-wise manner), an instantaneous disturbance compensation action, and (2) it induces, globally (i.e., in a total body-wise manner), a multi-dimensional force field with attractor dynamics. This allows achieving complex body postures “for free,” without a complex, high-dimensional computational process, but simply by allowing the intrinsic dynamics of the neuromuscular system to seek its equilibrium state.

In this framework, movement becomes the transition from an equilibrium state to another, with the remarkable property of “equifinality” (Kelso and Holt, 1980), namely the fact that movement end points should be scarcely affected either by small, transient perturbations or by variations in the starting position of the body. Such attractor properties of motor control were confirmed by several studies of electrical stimulation of different parts of the nervous system, such as interneurons in the spinal cord of the frog (Giszter et al., 1993) or pyramidal neurons in the precentral cortex of the monkey (Graziano et al., 2002).

The PMP (Mussa Ivaldi et al., 1988) was conceived as an extension of the EPH from motor control to motor cognition. The idea is to think that there are two attractor dynamics, nested one inside the other, which cooperate for action generation: the more internal one expresses an endogenous brain activity, related to an internal model or body schema and is the one that is responsible for covert movements (as such, it does not involve body masses, muscle stiffness, and muscle synergies); the latter attractor dynamics, related to the conventional EPH, exploits the physical equilibrium states determined by the biomechanics of the body and the neuromuscular system. Our hypothesis, in agreement with the Mental Simulation Theory (Jeannerod, 2001), is that the two dynamical regimes are compatible and integrated in the same structure, allowing subjects to shift effortlessly from mental simulations of actions to real actions and back.

**Figure 2:**
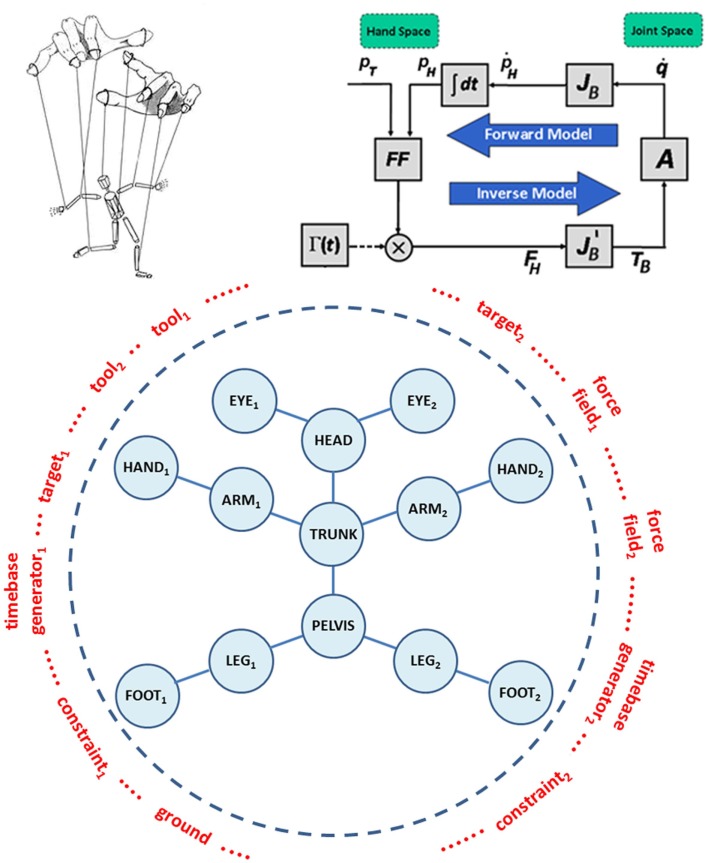
**Top panel – left part**: the animated marionette metaphor of the PMP-based body-schema concept. **Top panel – right part**: simplified example of a PMP network limited to the upper limb. *p*_T_ is the vector that identifies the 3D position of the Target in “hand space”; *p*_H_ is the corresponding position of the Hand; *FF* is the force-field generator; Γ(*t*) is the time-base generator; *F*_H_ is the force field applied to the Hand; *T*_B_ is the high-dimensional, whole-body Torque field; *A* is the admittance matrix. The basic kinematic constraint that links the Hand and Joint spaces is represented by the Jacobian matrix of the body *J*_B_. Additional constraints, in the hand and joint spaces, can be represented by means of corresponding force or torque fields. **Bottom panel**: generalized representation of the whole-body schema within the PMP framework, to be configured in the preparation time of an action with a selection of *tools*, *targets*, *time-base generators, ground*, and specific *task-oriented constraints*.

Passive motion paradigm is a force-field-based mechanism of synergy formation that allows coordinating the motion of a redundant set of articulations while carrying out a task, like reaching or tracking an object. Originally, it was formulated in order to demonstrate that, when carrying out inverse kinematics with a highly redundant system, it is not necessary to introduce an explicit optimization process. The idea can be expressed, in qualitative terms, by means of the animated puppet metaphor (see Figure 2, top panel, left part): the puppet is a bare skeleton called to “life” by the puppeteer by applying a small set of force fields to critical end-points, specific for a given task or gesture. The key issue is that in the animation of the action it is not the proximal part of the body, which is pushing the end-effector to the target, but the other way around: the end-effector is pulled toward the target by the force field and in turn pulls the rest of the body.

The right part of the top panel in Figure 2 shows a simplified example of a *PMP network* or block diagram, limited to the upper limb. In mathematical terms, the block diagram can be described as follows: the intention to reach a target *p*_T_ with the hand is represented by a force field *F*_H_, aimed at the target and attached to the hand *p*_H_. *F*_H_ is mapped into an equivalent torque field *T*_B_, acting on all the joints of the arm (vector *q*), by means of the transpose Jacobian matrix $J_{B}^{T}:$ it is worth noting that the torque field has a much higher dimensionality than the force field as a consequence of the redundancy of the arm. The torque field induces in the body schema a distribution of incremental joint rotations, modulated by the admittance matrix *A*. In turn, the joint rotation pattern is mapped into the corresponding hand motion pattern, thus updating the attractor force field and closing the computational loop. It should be noted that all the computations in the loop occur in parallel and are “well posed,” thus the computational model is robust and cannot fail, whatever the degree of redundancy. The time for reaching the new equilibrium from the initial one is determined by using a technique proposed by the group of Zak (1988), called *terminal attractor dynamics*: it consists of a non-linear modulation of the force field, which tends to diverge to infinity when time approaches the intended deadline. The function Γ(*t*) or non-linear time-base generator (TBG) implements such modulation. The function can be considered as a kind of “neural pace-maker” (Barhen et al., 1989) and a biologically plausible representation can be identified in the cortico-basal ganglia-thalamo-cortical loop and the well-established role of the basal ganglia in the initiation and speed–control of voluntary movements.

The PMP network described above is a dynamical model of synergy formation, which is characterized by attractor dynamics, in agreement with EPH. It is also multi-referential, because dynamical systems are intrinsically parallel, without any hierarchy between low-dimensional hand space and high-dimensional joint space. In this specific example, it tends to generate straight hand trajectories because it is driven by a force field applied to the hand, as it happens for reaching movements in free space. However, the same type of multi-referential network might generate different types of trajectories if the target was defined in the joint space or if other force fields were added in order to introduce task-dependent constraints or obstacles.

In the general case of whole-body movements, the PMP network of the body is depicted in the bottom panel of Figure 2. The network includes the skeleton of the whole body (blue part), which needs to be initialized and specialized for each intended action by dynamically linking it to a number of modules (marked in red): “targets,” “force fields,” “TBGs,” “tools,” “ground,” etc. For example, the PMP network in the top panel is virtually produced as follows: a target and the corresponding force field are linked to the right hand; the right shoulder is “grounded,” meaning that the force field applied to the hand can be propagated through the arm, terminating in the shoulder; the TBG is linked to the force field and then the animation is initiated, generating a coordinated movement of the right arm until the target is reached, if it is indeed reachable, or the arm is fully stretched in the direction of the target, if the target is outside the workspace. This means that each action generated according to this computational model always involves, potentially, the whole body but simpler synergies, related to a smaller number of degrees of freedom, can be obtained by “grounding” specific parts of the body schema, without requiring separate models for specific gestures.

The *blue part* of the animated body schema can be associated to proprioception: it stores the *proprioceptive image* of the body; the *red modules* may correspond to visual, auditory, or even olfactory signals. The red modules are recruited dynamically according to the task, whereas the blue part of the animated body schema is always involved. We may summarize this concept by saying, as already observed in Section “Coding and Frames of Reference in Sensorimotor Cortical Maps: The Body-Schema is Dynamic and Multi-Referential,” that proprioception is the “glue” that keeps the coherence of the body schema.

In general, *syn-ergy* formation requires a *sym-phonic* director, not a mere metronome, but a coordination entity that, in addition to giving the tempo, recruits the different sections of the orchestra, modulates the emphasis of the different melodic pieces, etc.: the gating action of the TBG is the key element of this symphonic action. In this view, the role of motor cognition is to prepare and initialize the body schema by selecting and tuning the appropriate *red modules*, exploiting learned and memorized patterns that are likely to be the best in a given context. The final act of such cognitive preparation is to start the animation by triggering the TBG.

The movements determined by the PMP model are described as “passive” in the sense that it is passive that the animation of a marionette: the joint rotation patterns are not explicitly programed, but are the consequences of applying a set of virtual forces to the terminal parts of the virtual marionette. It is worth mentioning that the body-schema model implemented by the computational block diagram of Figure 2 generates target-oriented movements that satisfy the already described spatio-temporal invariants, such as the bell-shaped speed profile, without any explicit optimization process. Moreover, the “admittance” matrix *A*, specifies the degree of participation of each joint to the common reaching movement, and thus it can be modulated according to specific task requirements solving in this manner the redundancy problem.

The body schema is embedded in the Jacobian matrix and the model of the task in the force-field generators and the admittance matrix. The network can be easily generalized to whole-body movements, which recruit all the DoFs of the body, can be expanded in order to integrate manipulated tools, and can be easily specialized to a variety of tasks, even multiple, concurrent tasks. In the PMP framework, force fields, admittance, and stiffness matrices do not refer to physical entities, as happens in the classical EPH framework, but to features of the attractor dynamics of the internal body model.

We already explained in which sense the proposed model is intrinsically multi-referential. We explain here in which manner it is also multi-tasking and/or multi-constraint. This property is “inherited” by the fact that the PMP-based model is a member of the EPH-family, namely is based on force field as basic computational elements, which operate in parallel. In this framework, movements are not explicitly programed but are the consequences of simulating a dynamic system activated by a set of force fields. The power and generality of the concept are that in a complex, redundant, articulated system like the human body elementary/component force fields can always be superimposed, whereas elementary/component movements cannot.

A similar point of view has been followed by Kutch and Valero-Cuevas (2012) in their analysis of muscle synergies, but with a different conclusion: they show that the biomechanics of the limbs constrain musculo-tendon length changes to a low-dimensional subspace across all possible movement directions and then propose that “a modest assumption” (*that each muscle is independently instructed to resist length change*) can explain the formation of neuromuscular synergies. The “modest assumption” above is equivalent to the “passive motion” of the PMP model. However, the conclusion by the former authors (namely, that *muscle synergies will arise without the need to conclude that they are a product of neural coupling among muscles*) is not the only possible one. The alternative, exemplified by the PMP hypothesis, is that the neural coupling (or the organized S-state, borrowing the terminology of Jeannerod, 2001) is just the result of the simulation of the passive motion induced by the internal body model.

The PMP-based body schema illustrated in this section is “muscleless” and “massless”^5^. Its animation will generate sensorimotor patterns that are not directly linked to the control of an overt action but to the “imagination” of the corresponding covert action. However, we know from brain imaging studies that overt and covert actions must share a common format and if we accept the animated body schema as a plausible mechanism for the generation of covert actions, we should also accept the hypothesis that such simulation patterns are somehow used also in the motor control of the overt actions. However, we should point out that this hypothesis does not imply the simplistic view, sometimes adopted by roboticists, that motor control consists in simple tracking of reference trajectories derived from a motor plan. Rather, we posit that the link between motor plan and motor control is not univocal and unidirectional but, on one hand, may require a choice among multiple control strategies and, on the other hand, a bidirectional interaction between cognition and control. However, the discussion of this point is outside the scope of this paper. This is just to explain in which sense we think that motor cognition and motor control are separate processes but communicate bidirectionally through the animated body schema. Ultimately, we should include higher levels of motor cognition, that are involved, for example, in the utilization of different forms of memory for accumulation of motor knowledge, because this is likely to enrich the skillfulness of the body schema (Mohan et al., 2014).

## The Body-Schema and Whole-Body Reaching

In order to illustrate the organization and the potentialities of the PMP-based body schema, let us consider the task of reaching and lifting a load with the whole body in a bipedal standing condition. A preliminary formulation is described in Morasso et al. (2010). It is limited to the sagittal plane and it involves only five DoFs (ankle, knee, hip, shoulder, elbow), with the goal of generating kinematic patterns of a five DoFs stick figure in the context of a double task: (1) a *focal task* (reaching or approaching as much as possible a target in the sagittal plane); (2) a *postural task* [keeping the vertical projection of the center of mass (CoM) inside the support base of the standing body]. The formulation, which is described here, is a three-dimensional extension, which involves 32 degrees of freedom (Morasso et al., 2014) and also incorporates additional internal and external constraints. In particular, the model is under the action of five force/torque fields that identify the focal-postural tasks and biomechanical/physiological constraints:

1. *Focal field* (*FF*_foc_): it is applied to the hand and pulls it to the (moving) target. It is an attractive field.
2. *Postural field* (*FF*_pos_): it is applied to the pelvis and aims at keeping the projection of the CoM inside the support base, i.e., inside the interval *x*_min_ ↔ *x*_max_. It is a repulsive field.
3. *ROM field* (*FF*_rom_): it repulses each joint from the designated joint limits (*q*_min_ ↔ *q*_max_) in order to assure that joint rotation patterns generated by the model are consistent with the physiological RoM (range of motion) of each joint.
4. *Head gaze field* (*FF*_gaze_): it is a “head-to-target field” or “visual focal field,” which attempts to keep the gaze direction aimed to the target by inducing appropriate rotations of the cervical joint.
5. *Neck vestibular field* (*FF*_ves_): it simulates a “vestibular” stabilization of the neck, with the goal to keep the neck approximately vertical.

In summary, the overall dynamics of the PMP-based body schema is represented by the following set of non-linear differential equations:

(1) $$\begin{array}{l} \left\{\;\begin{array}{cc} {\dot{p}}_{T}=\Gamma \left(t\right)\left(p_{F}-p_{T}\right)\; & \mathrm{Moving target generation:}p_{T}\left(t\right)\to p_{F}\;\\ F_{\mathrm{foc}}=K_{e}\left(p_{T}-p_{H}\right)\; & \mathrm{Calculation of the focal force field}\;\\ T_{\mathrm{foc}}=J_{e}^{T}F_{\mathrm{foc}}\; & \mathrm{Calculation of the focal torque field}\;\\ T_{\mathrm{pos}}=J_{p}^{T}F_{\mathrm{pos}}\left(q\right)\; & \mathrm{Calculation of the postural field}\;\\ T_{\mathrm{RoM}}=T_{\mathrm{RoM}}\left(q\right)\; & \mathrm{Calculation of the RoM field}\;\\ T_{\mathrm{gaze}}=T_{\mathrm{gaze}}\left(q\right)\; & \mathrm{Calculation of the head gaze field}\;\\ T_{\mathrm{neck}}=T_{\mathrm{neck}}\left(q\right)\; & \mathrm{Calculation of the neck vestibular field}\;\\ T_{\mathrm{total}}=T_{\mathrm{foc}}+T_{\mathrm{pos}}+T_{\mathrm{neck}}+T_{\mathrm{head}}+T_{\mathrm{RoM}} & \mathrm{Calculation of the total field}\\ \dot{q}=\Gamma \left(t\right)AT_{\mathrm{total}}\; & \mathrm{Relaxation of the body-schema}\to q\left(t\right)\;\\ \; \end{array}\right. \end{array}$$

The body schema implemented by this mathematical model is clearly multi-referential and it mixes external/internal constraints in different spaces: *FF*_foc_ and *FF*_pos_ are defined in the Cartesian space, *T*_RoM_ and *T*_neck_ in the joint space, and *T*_gaze_ in the visual and joint spaces. The two Jacobians (*J*_e_ and *J*_p_) propagate the focal and postural force fields to the appropriate kinematic chains (hand to foot, in the former case, and pelvis to foot, in the latter).

According to the logic of the PMP model, the different force or torque fields are superimposed in the joint space, providing a total torque field, for each joint of the body schema. Thus, in principle each movement is always a whole-body movement, even if the task is focused on active coordination of a small subset of joints. However, it is possible to specialize the PMP model by “grounding” sections of the body schema, in such a way that task-related force fields can propagate only to a subset of joints and thus limit synergy formation only to that subset.

In any case, the total torque field is “gated” by the admittance array *A*, thus inducing a corresponding array of joint rotation speeds $\dot{q}.$ The relative values of the admittance array can be modulated in order to emphasize or depress the degree of participation of any joint to the common task. Thus, *A* is a task-dependent parameter that can be exploited in order to express “cognitive requirements/constraints.” For example, let us suppose that a subject is trained to perform a given gesture by almost “freezing” the motion of a joint and greatly “enhancing” the mobility of another joint: this is simply implemented in the PMP model by depressing the admittance of the former joint and amplifying the admittance of the latter, in relation with the standard admittance values of the other joints.

The mathematical model is a dynamical system where all the computations listed above occur in parallel, presumably in different cortical areas coordinated in time by a sub-cortical TBG or “neural pace-maker,” corresponding to the Γ function above. Thus, there is no explicit control of timing but temporal coordination is indirectly provided by the TBG, which is responsible for the initiation of the overall dynamics and its termination at the preset temporal deadline. In the mathematical model above, the TBG drives at the same time the generation of the moving target for the hand and the relaxation to equilibrium of the whole body. Moreover, the TBG can be triggered several times in a row, in order to implement sequential tasks, with several sub-goals, as the box grasp and lift task: reach, grasp, and lift. Some overlap is also possible between one activation and the next one of the synergy formation machinery as it happens in smooth complex gestures in sport or dance.

It is worth noting that the same Jacobian matrix that, in the transposed version, is used for transforming the attractive force field applied to the hand into the corresponding torque field and is also the operator that maps the joint speed vector into the corresponding hand velocity vector. A key feature of this attractor dynamics is that although the force field attracts the hand to the target in a straight manner, the trajectory followed by the hand may be quite curved because, in general, the eigenvectors of the matrix that characterize the overall transformation from the joint to the hand space will not be oriented as the force field. For this reason, for reaching tasks it is convenient to use a “moving target,” which slides smoothly from the initial position of the hand to the final target position, a feature supported experimentally by Bizzi et al. (1984) when they suggested the concept of “virtual trajectory control.”

The key elements of the model, such as the Jacobian matrices, force/torque fields, or the TBG, do not need to be neurally represented in a direct way but are indirect features of the overall dynamics. The coherence of such dynamic organization is not established once for all but must be refreshed/reestablished in the course of action and adaptation to a changing body and changing environment. Keeping the body schema updated and in touch with “reality” is, in a sense, the main purpose of the body schema itself, in agreement with the principles of autopoiesis (Maturana and Varela, 1980).

### Simulation of the body-schema model for the reach and lift task

Starting from a relaxed standing posture, the task involves two subtasks:

(1) reaching the target object and
(2) lifting it up.

**Figure 3:**
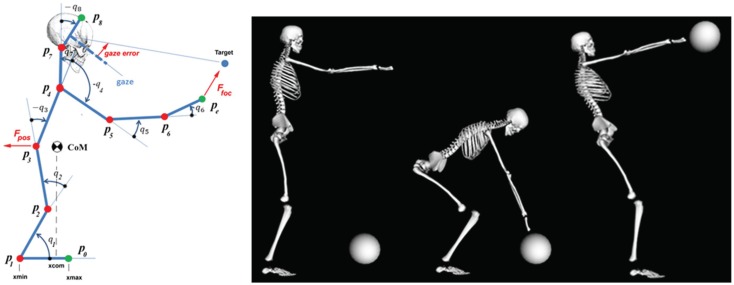
**Animation of the body schema for the ball grasp and lift task**. *Left panel*: stick figure related to the simulation. q1: ankle joint; q2: knee joint; q3: hip joint; q4:shoulder joint; q5: elbow joint; q6: wrist joint; q7: neck joint; q8: cervical joint. *Right panel*: key postures of the reach and lift task.

**Figure 4:**
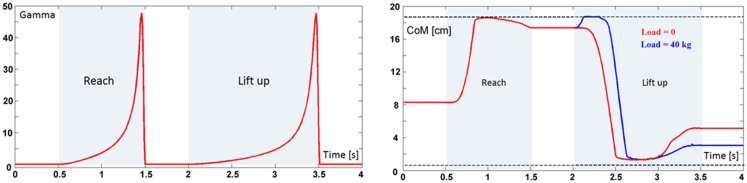
**Left panel**: time-base generator (Gamma function) triggered twice in the reach and lift task. **Right panel**: evolution of the forward/backward component of the vertical projection of the body CoM on the standing surface during the reach and lift task, for two loads, namely 0 kg (red trace) and 40 kg (blue trace). The dashed lines correspond to the limits of static stability (*x*_min_ and *x*_max_, respectively). The medio-lateral component can be neglected because this simulation is limited to in the sagittal plane.

**Figure 5:**
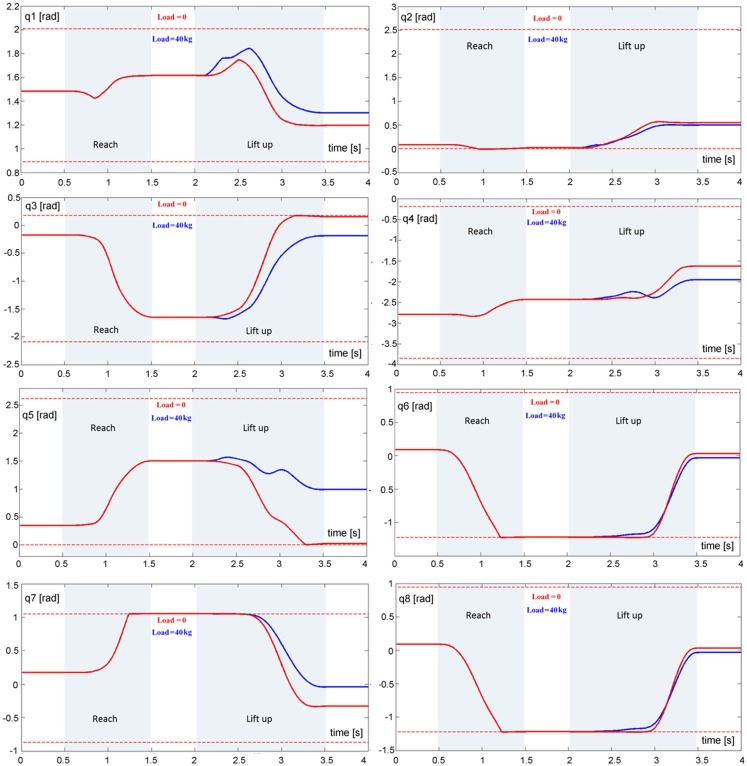
**Evolution of eight DoFs of the model during the reach and lift task for two loads, namely 0 kg (red trace) and 40 kg (blue trace): q1 (ankle joint), q2 (knee joint), q3 (hip joint), q4 (shoulder joint), q5 (elbow join), q6 (wrist joint), q7 (neck joint), and q8 (cervical joint)**. The dashed lines correspond to the limits of the RoM for each joint.

The left panel of Figure 3 shows the stick figure. Although the simulation refers to movements in the sagittal plane, the underlying model is 3D and relates to the full set of degrees of freedom. The right panel of the figure shows the initial posture, the posture at the end of the reaching subtask, and at the end of the lifting subtask, for a 40 kg ball. Figures 4 and 5 show the results of the simulation of the body-schema model, configured for the reach and lift task, which consists of integrating the set of equations (1). For simplicity, the simulations are limited to the sagittal plane but the model is fully three-dimensional. The simulation is “massless,” except for the postural force-field generator that requires the on-line computation of the projection of the body CoM on the standing surface.

The TBG is triggered twice in this task (Figure 4, left panel): the first activation (duration = 1 s) drives the reaching movement and the second activation (duration = 1.5 s) drives the lift up movement.

It is not easy to find realistic anthropometric and biomechanical parameters to be used in model simulation. They are scattered in a number of publications/reports (Herron et al., 1976; Roaas and Gunnarb Andresson, 1982; Zatsiorsky and Seluyanov, 1983; De Leva, 1996; NASA-STD-3000, 2000; Pavol et al., 2002; Hersch and Billard, 2008; Paquette et al., 2009). The following simulations were carried out by extracting data from the mentioned literature, summarized in Morasso et al. (2014). Figure 4 (right panel) shows the evolution of the CoM during the entire task, demonstrating that the synergy formation process satisfies the constraints of static stabilization while succeeding to reach the target positions. Moreover, Figure 5, which shows the evolution of the joint angular rotations for eight joints of the model during the entire task, demonstrates that also the joint rotation patterns satisfy the RoM constraint in a smooth way. It is also possible to show that the requirements formulated for the gaze and neck by the corresponding force field are smoothly implemented in the course of the synergy formation process, in parallel to the trajectory of the end-effector to the target and the synchronized motion of the CoM inside the prescribed range of stability.

By analyzing the kinematics of the end-effector for generic reaching tasks, it is possible to demonstrate that the simulation of the model is capable of reproducing well-known spatio-temporal kinematic invariants, as the bell-shaped speed profile, without any explicit specification of a desired time course of the trajectory or utilization of optimization process. In the specific case of the focal-postural task, if we consider the kinematics of the CoM of the whole body, we can verify that the same model is capable to predict additional less known invariant features: (1) the forward shift of the CoM and (2) the synchronized velocity peaks of the reaching hand and the CoM shift (Pozzo et al., 2002; Kaminski, 2007). Since such features are not imposed by the task or by biomechanical constraints, they are likely to reflect the organization of the synergy formation process. Thus, the prediction by the body-schema simulation of the specific coordination between the end-effector and the CoM is verified by experimental evidence. This also suggests that the observed coordinated patterns are mainly explained by the intrinsic dynamics of the “massless/muscleless” body schema rather than by the adoption of a specific control strategy.

**Figure 6:**
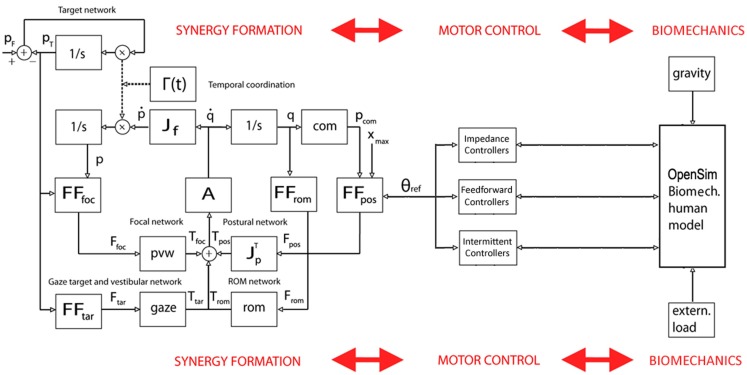
**Block diagram of the PeterPan simulation carried out for the grasp and lift task**. θ_ref_ is the output of the synergy formation process, driven by the PMP-based body schema, and the input to the set of motor controllers, which interact with the OpenSim biomechanical model. In general, the interactions are bidirectional in order to allow adaptation to unpredicted environmental conditions or changing task requirements.

More recent experimental and theoretical investigations provide further support to the rationale of the modeling framework described in this paper. Fautrelle et al. (2010) showed that equilibrium constraints do not affect the timing of synergies during whole-body reaching movements, suggesting a higher coordination level of synergy formation, similar to the PMP mechanism. Chiovetto et al. (2012) found that reversing the direction of whole-body reaching movements does not affect the level of joint co-variation, although the differential influence of gravity (in upward vs. downward movements) was clearly detected in the muscle activation patterns. In general, the modular organization of the proposed body schema is quite in tune with the modular control of pointing beyond arm’s length described by Berret et al. (2009). Moreover, although we did not investigated in a detailed manner how to configure the model of Figures 2 and 6 in order the include the coordination of pointing during locomotion, we expect to obtain coordinated reaching and walking movements that exhibit reciprocal patterns of influence similar to those described by Chiovetto and Giese (2013). In this case, we would need to introduce a central pattern generator (CPG) alongside the previously considered TBG.

What the simulation of the PMP-based body schema cannot predict is the behavior of the standing body at the beginning or after the end of a whole-body reaching movement. Since this model is characterized by attractor dynamics, the initial and the final postures are equilibrium configurations of an asymptotically stable multi-dimensional system. In contrast, posturographic analysis of the upright body in quiet standing shows persistent sway patterns, which can only be interpreted by the interaction of the body schema with an underlying control system, operating according to a specific control strategy and interacting with the biomechanics of the body. As a consequence, if the body-schema model is going to be used in order to make predictions about motor coordination in general, it is necessary to extend it with a model that includes the dynamics of the body and the control strategies, as shown in the following section.

## Whole-Body Postural-Focal Dynamics

This postural force field *FF*_pos_ considered in the previous section implements a *hip strategy* of static stabilization, where “static” refers to the fact that the proprioceptive image of the whole body, generated by the animation of the body schema, for each time instant of the simulation is capable to keep the vertical projection of the CoM on the standing surface inside the support base. This stability constraint also applies to the initial and the final posture of each whole-body movement and, as already observed in the previous section, it should imply fixed equilibrium postures but this is in contrast with the observed sway movements, a kind of uncontrolled oscillation of small amplitude that characterize quiet standing. As a consequence, the static stabilization provided by the focal-postural activation of the body schema in whole-body reaching movement is only a necessary condition of stability of the standing posture. It would be also sufficient in the case of a stiffness control strategy (hypothesized by Winter et al., 1998), which is based on the hypothesis that ankle stiffness is greater than the rate of growth of the toppling torque due to gravity. In this case, the overall dynamics of the body would be characterized by point attractor dynamics and the residual sway patterns could only be attributed to neuromuscular noise.

This hypothesis was falsified by direct stiffness measurements (Loram and Lakie, 2002; Casadio et al., 2005), which demonstrated that ankle stiffness is insufficient for achieving asymptotic stability of the standing body. As a consequence, a *dynamic stabilization loop* must operate alongside the *static stabilization synergy formation mechanism* described above. In a subsequent paper (Asai et al., 2009), it was demonstrated that in quiet standing physiological sway patterns can be explained by a *non-linear feedback controller based on intermittent feedback*, namely a switching mechanism that exploits the peculiar *saddle-like dynamic instability* of the bodily inverted pendulum. In this framework, the dynamic stabilization of the standing body is not characterized by a point attractor, disturbed by high-level noise, but by a kind of quasi-periodic attractor, or limit cycle, perturbed by a much lower level of noise.

However, the efficacy and biological plausibility of the intermittent feedback controller (Asai et al., 2009) was only demonstrated in the case of quiet standing, in which the movements of the body, with good approximation, can be drastically reduced to a single DoF system, namely the inverted pendulum model. On the other hand, there is no guarantee that this control mechanism is robust enough to stabilize the standing body also in 3D whole-body movements of large size, involving a large number of degrees of freedom. This is a typical example in which it is quite useful to have a composite simulation package that combines a body-schema model and a biomechanical model of the body dynamics, in order to investigate the link between motor cognition and motor control.

For this reason, we started the development of a simulation package that has been named *PeterPan* (Morasso et al., 2014) in order to emphasize the bidirectional *cognitive* interaction between Peter Pan (the *body*) and his shadow (the *body-schema*). *PeterPan* includes (1) the PMP-based body schema described in the previous section; (2) a biomechanical simulator of whole-body dynamics based on OpenSim/Simbody^6^; (3) a set of neuromuscular controllers for the different DoFs; (4) a software middleware YARP^7^ for integrating in a robust way the different parts of the simulation package.

The first application of *PeterPan* is the preliminary analysis of whole-body postural-focal dynamics. Figure 6 shows a simplified block diagram of the main modules. In particular, the following set of neuromuscular control modules was used: (1) feedforwad compensation of gravity for all the joints, taking the output of the body schema as a generator of whole-body reference postures; (2) intermittent dynamic stabilization of the ankle joint, with the same control parameters used by Asai et al. (2009) but with a variable moment of inertia; (3) impedance control of the all the other joints around the reference trajectories. A small amount of noise is injected into the system in order to take into account the postural disturbance induced by the heartbeat and muscle noise (Conforto et al., 2001).

**Figure 7:**
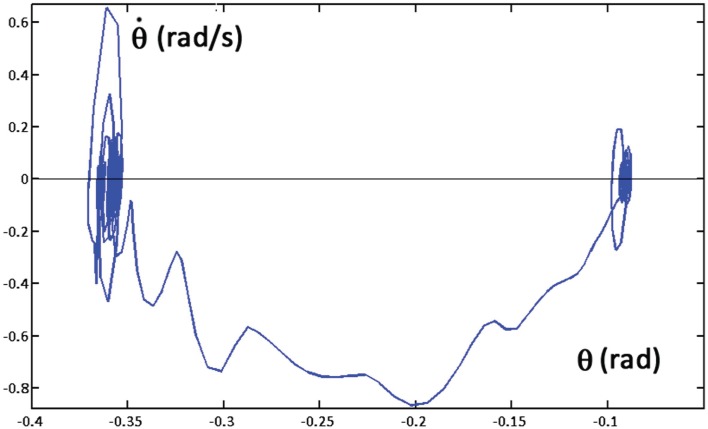
**Sway movements generated by the *PeterPan* simulation package during a whole-body reaching movement: sway angle θ vs. sway speed dθ/dt**. The initial posture (tilted forward 0.1 rad) corresponds to the oscillations on the right part of the graph; the final posture (tilted forward 0.37 rad) to the oscillations on the left part of the graph. The ragged curve corresponds to the shift from the initial equilibrium to the final equilibrium posture.

The already mentioned Figures 4 and 5 illustrate the coordinated motor patterns generated by the synergy formation mechanism based on the body schema, i.e., the integration of the set (1) of differential equations. In particular, Figure 4 (right panel) shows that *static stabilization* is achieved directly by the body schema because the coordinated motion patterns maintain the virtual evolution of the CoM inside the admitted support base. The simulation of the overall system depicted in Figure 6 (PMP + Control Modules + OpenSim biomechanical simulator) generates coordinated motor patterns similar to those of Figures 4 and 5, although slightly different due to partially unaccounted dynamic effects and the above-mentioned postural noise. In particular, let us focus on the dynamic stability of the model. As shown in the previous Section “Simulation of the Body Schema Model for the Reach and Lift Task”, the configured body schema incorporates the constraint of static stability, but dynamic stability is challenged by the fact that in agreement with biological evidence the stiffness of the ankle joint, implemented in the OpenSim model, is insufficient to enforce asymptotic stability of the overall system. Nevertheless, the simulation of the overall system demonstrates the robust bounded stability of the reach and lift gesture. In particular, Figure 7 shows the sway patterns (sway angle vs. sway angular velocity) of the whole body during a whole-body reaching movement, resulting from the interplay among synergy formation, motor control, and biomechanics. The figure demonstrates that the posited distribution of motor control actions succeeds to achieve *dynamic stabilization* on top of the *static stabilization above*. Moreover, the spatio-temporal analysis of the sway patterns is compatible with posturographic data.

In general, a simulation package like *PeterPan* can be used for formulating hypotheses about the interaction between motor cognition and motor control in such a way to make predictions about specific motor patterns to be verified in corresponding experiments. We believe that both neuroscience and cybernetics need a robust formulation of embodied cognition based on the concept of body schema. This will allow us to better understand what action is, in the most general sense, linking experimental evidence coming from a large variety of approaches and experimental setups. The *PeterPan* simulation package provides a tool in this direction.

## Conflict of Interest Statement

The authors declare that the research was conducted in the absence of any commercial or financial relationships that could be construed as a potential conflict of interest.

## Footnotes

^1^Such apparently “solid” coherence is violated by “revealing mishaps” as the famous rubber hand illusion (Lewis and Lloyd, 2010).

^2^This concept if further explained in Section “A Formulation of the Body Schema Based on the PMP as a Synergy Formation Mechanism” of the paper.

^3^Inverse dynamics, i.e., transforming desired kinematic patterns into the corresponding torque patterns, should not be confused with inverse muscular dynamics, i.e., transforming the torque profiles into corresponding muscle force profiles. The latter process is ill-posed whereas the former one is not.

^4^Motor cognition has clearly different connotations in different fields. In the context of this paper, we use the following operational definition of (embodied) motor cognition: the mental process involved in the preparation, imagination, and production of actions with the purpose of initializing and specializing the body schema in such a way to animate it in a task-oriented manner.

^5^The PMP-based body schema is “muscle-less” and “mass-less” in the sense that its animation/simulation does not generate muscle forces and/or joint torques that depend on the distribution of masses on the body segments. It is a synergy formation mechanism that combines a number of abstract force fields, intended to express internal and external constraints, independent of muscle biophysics and whole-body body Lagrangian biomechanics. However, some force fields, such as the Postural Field described in the next section for whole-body reaching, imply the knowledge of the position of the vertical projection of the center of mass on the standing surface.

^6^https://simtk.org/home/opensim

^7^http://wiki.icub.org/yarpdoc/
